# CSF Anti-Sjögren’s Syndrome Antigen A Antibodies and Low Glucose Levels in Sjögren’s Syndrome-Associated Aseptic Meningitis: A Case Report

**DOI:** 10.7759/cureus.103059

**Published:** 2026-02-05

**Authors:** Tatsuya Ueno, Ko Hiyama, Ren Yanagida, Maki Miura, Akira Arai

**Affiliations:** 1 Neurology, Aomori Prefectural Central Hospital, Aomori, JPN

**Keywords:** anti-ro antibodies, anti-ss-a antibodies, aseptic meningitis, central nervous system, hypoglycorrhachia, sjögren’s syndrome

## Abstract

Primary Sjögren’s syndrome (pSS) rarely involves the CNS, including cases of aseptic meningitis (AM). We report the case of a 59-year-old woman who presented with headache, fever, and nuchal rigidity. CSF analysis revealed pleocytosis, elevated protein, and decreased glucose levels. Both serum and CSF anti-Sjögren’s syndrome antigen A (anti-SS-A) antibody tests were positive, with an elevated CSF anti-SS-A antibody index. Salivary gland biopsy confirmed the diagnosis of pSS. The patient improved following steroid pulse therapy. Reduced CSF glucose levels in combination with positive CSF anti-SS-A antibody tests may serve as supportive clues for pSS-associated AM. Evaluation of autoimmune features and CSF anti-SS-A antibodies may aid in the diagnosis of AM of unclear etiology.

## Introduction

In primary Sjögren’s syndrome (pSS), CNS involvement occurs in 14-19% of cases, manifesting as myelitis, encephalopathy, psychiatric symptoms, seizures, and aseptic meningitis (AM) [1]. AM associated with pSS is clinically important because it can mimic infectious meningitis, often leading to diagnostic uncertainty and delays in appropriate immunosuppressive treatment.

Anti-Sjögren’s syndrome antigen A (anti-SS-A) antibodies have been proposed as a pathogenic factor in SS-associated CNS disorders [2], potentially contributing to meningeal inflammation through immune-mediated mechanisms, including intrathecal antibody production, blood-brain barrier disruption, and antibody-associated inflammatory responses within the CNS [2,3].

However, to our knowledge, anti-SS-A antibodies in the CSF of patients with AM have been reported in only one published study [4]. Recent evidence suggests that autoimmune AM can occur in pSS even in the absence of anti-SS-A antibodies [5]. Thus, the clinical significance of CSF anti-SS-A antibodies and their association with specific CSF profiles, including hypoglycorrhachia, remain incompletely understood.

We report a case of pSS-associated AM characterized by positive findings for serum and CSF anti-SS-A antibodies, pleocytosis, and notably decreased CSF glucose levels. To our knowledge, this is among the few reported adult cases demonstrating both an elevated CSF anti-SS-A antibody index and hypoglycorrhachia in pSS-associated AM, potentially providing further insight into this rare manifestation.

## Case presentation

A 59-year-old woman presented to the emergency department with a nine-day history of headache and arthralgia predominantly affecting her large joints, a seven-day history of fever up to 38°C, and a four-day history of poor oral intake. She had experienced xerostomia three years prior and tested positive for anti-SS-A antibodies. Her medical history included ischemic colitis and allergic reactions to contrast media.

On admission, her Glasgow Coma Scale score was 15. Vital signs were as follows: body temperature of 37.1°C, blood pressure of 124/79 mmHg, heart rate of 79 beats/min, respiratory rate of 16 breaths/min, and oxygen saturation of 98% on room air. A general physical examination revealed no evidence of arthritis or lymphadenopathy. Her neurological examination was unremarkable except for nuchal rigidity; cranial nerve function, motor strength, sensation, coordination, and reflexes were all normal.

Laboratory studies showed normal blood counts with a mildly elevated CRP level (0.73 mg/dL). Autoantibody testing revealed an antinuclear antibody titer of 1:320 with a speckled pattern and strongly positive anti-SS-A (>1200 U/mL), while anti-SS-B and other autoantibodies were negative.

CSF analysis demonstrated a cell count of 179 cells/mm³ (93.9% mononuclear cells), protein 96 mg/dL, glucose 39 mg/dL (serum glucose 121 mg/dL), adenosine deaminase 11.0 U/L, angiotensin-converting enzyme 0.4 U/L, IL-6 26.5 pg/mL, albumin quotient 12.7 × 10⁻³, IgG index 0.61, and a negative oligoclonal band test. The CSF anti-SS-A antibody index was elevated at 6.40 (Table 1).

**Table 1:** Laboratory parameters anti-dsDNA, anti-double-stranded DNA antibody; anti-RNP, anti-ribonucleoprotein antibody; anti-Scl-70, anti-scleroderma 70 antibody; anti-Sm, anti-Smith antibody; MPO-ANCA, myeloperoxidase-antineutrophil cytoplasmic antibody; PR3-ANCA, proteinase 3-antineutrophil cytoplasmic antibody; Qalb, albumin quotient (CSF/serum albumin ratio)

Parameters	Patient values	Reference range
Serum		
White blood cells (/μL)	6.4 × 10³	3.3-8.6 × 10³
Hemoglobin (g/dL)	12.5	11.6-14.8
Platelets (/μL)	2.19 × 10⁵	1.58-3.48 × 10⁵
CRP (mg/dL)	0.73	0.0-0.14
Antinuclear antibody	1:320, speckled pattern	-
Anti-SS-A antibody (U/mL)	>1200	-
Anti-SS-B antibody	Negative	-
Anti-dsDNA antibody	Negative	-
Anti-Sm antibody	Negative	-
Anti-RNP antibody	Negative	-
Anti-Scl-70 antibody	Negative	-
Anti-centromere antibody	Negative	-
MPO-ANCA	Negative	-
PR3-ANCA	Negative	-
Glucose (mg/dL)	121	73-109
CSF		
Color	Clear and colorless	-
CSF pressure (mmH₂O)	140	70-180
White blood cells (cells/mm³)	179	<5
Polymorphonuclear leukocytes (%)	11	-
Protein (mg/dL)	96	15-45
Glucose (mg/dL)	39	40-70
Adenosine deaminase (U/L)	11	<4
Angiotensin-converting enzyme (U/L)	0.4	<0.5
IL-6 (pg/mL)	26.5	<4.3
IgG index	0.61	<0.7
Qalb (×10⁻³)	12.7	<9
Oligoclonal bands	Negative	-
Anti-SS-A antibody index	6.4	<1.5

**Figure 1:**
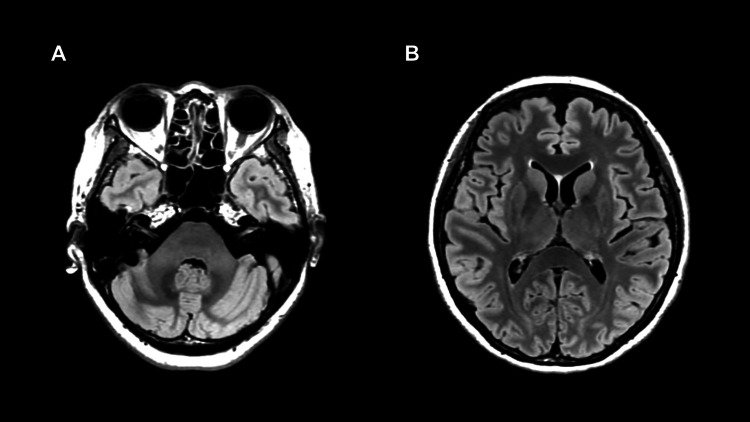
Brain MRI Axial brain fluid-attenuated inversion recovery MRI images. (A and B) The images show no apparent abnormalities. All imaging studies were performed before initiation of steroid therapy.

The meningitis/encephalitis panel and cultures were negative. Non-contrast brain MRI and whole-body CT showed no abnormalities (Figure 1).

**Figure 2:**
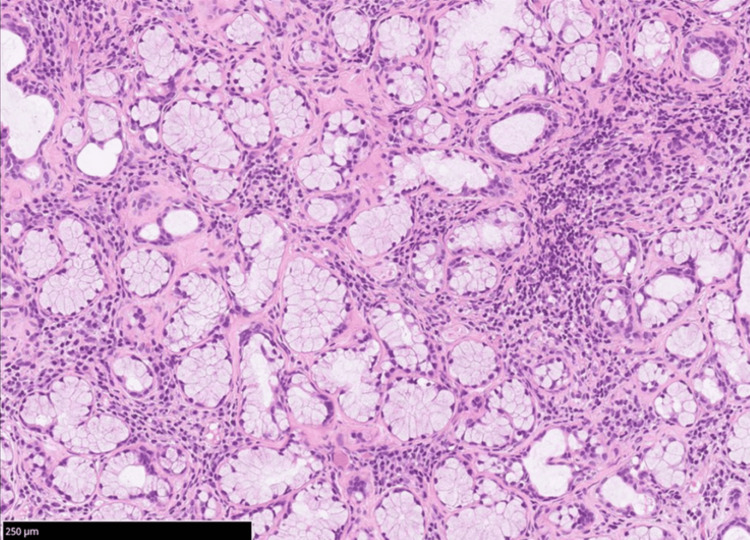
H&E staining Minor salivary gland biopsy demonstrating Greenspan Grade 4 inflammation. Scale bar = 250 μm.

The patient was admitted for AM. Empirical treatment with acyclovir (1500 mg/day) was initiated and discontinued on day 3 after negative panel results were confirmed. Salivary gland testing and scintigraphy revealed reduced salivary secretion, and a minor salivary gland biopsy demonstrated Greenspan Grade 4 inflammation (Figure 2).

Based on these findings and the absence of other identifiable causes, the patient was diagnosed with pSS-associated AM. During the diagnostic workup, her fever and general malaise gradually improved without immunotherapy. Steroid pulse therapy (methylprednisolone 1 g/day for three days) was initiated on day 8, resulting in clinical improvement, and she was discharged on day 14. A follow-up CSF examination on day 26 showed marked improvement, with a cell count of 13 cells/mm³ (100% mononuclear cells), protein 18 mg/dL, and glucose 53 mg/dL (serum glucose 100 mg/dL). During six months of outpatient follow-up, no clinical relapse was observed.

## Discussion

This case extends current knowledge on Sjögren’s syndrome-associated AM by demonstrating CSF anti-SS-A antibodies, blood-brain barrier disruption, hypoglycorrhachia, and steroid-responsive improvement, thereby providing integrated pathophysiological insights. In cases of AM of unknown etiology, evaluation for autoimmune features (sicca symptoms, sialadenitis, and arthralgia) and measurement of CSF anti-SS-A antibodies can aid diagnosis, with reduced CSF glucose levels serving as a potential supportive finding in pSS-associated AM.

The differential diagnosis of AM includes systemic inflammatory and autoimmune disorders such as neurosarcoidosis, neuro-Behçet’s disease, Sjögren’s syndrome, systemic lupus erythematosus, and granulomatosis with polyangiitis, as well as drug-induced AM (most commonly associated with nonsteroidal anti-inflammatory drugs, antibiotics, intravenous immunoglobulin, and monoclonal antibodies) and neoplastic meningitis [6].

In a previous study of patients with pSS presenting with neurological manifestations, CSF pleocytosis was observed in 16 of 44 (36.3%) patients, and serum anti-SS-A antibodies were positive in 48.4% of patients with CNS involvement [7]. Another study examining 33 cases of SS-associated AM, or aseptic meningoencephalitis, reported that approximately 70% of cases occurred before or concurrently with SS diagnosis, with serum anti-Ro/SS-A antibodies present in 82% of cases [1]. A report of three cases of pSS with CNS involvement revealed serum and CSF anti-SS-A antibodies in all cases and anti-SS-B antibodies in one case. Intrathecal IgG production was suggested by elevated IgG indices or oligoclonal bands in all cases and by an elevated anti-SS-A antibody index (>1.5) in two cases [3]. Similarly, in two cases of SS presenting with cerebellar degeneration and peripheral neuropathy, CSF anti-SS-A antibodies were identified as potential biomarkers of CNS involvement [2]. The Ro52/tripartite motif protein 21 antigen, recognized by anti-SS-A antibodies, is widely distributed throughout the mouse CNS, with particularly high expression in Purkinje cells [2].

CNS involvement in pSS has been associated with high-titer anti-SS-A antibodies and extraglandular manifestations, suggesting systemic immune activation [7]. Additionally, elevated CSF anti-SS-A antibody indices in patients with pSS-related CNS involvement support intrathecal immune activation [3]. Although definitive risk factors remain unestablished, severe immune-mediated inflammation and extraglandular disease may predispose patients to CNS manifestations.

To our knowledge, only three prior reports comprising seven patients have revealed an association between CSF anti-SS-A antibodies and SS-related CNS involvement, with one patient presenting with AM (Table 2) [3,4,8].

**Table 2:** Characteristics of CNS involvement in Sjögren’s syndrome with CSF positive for anti-SS-A antibodies AI, anti-SS-A antibody index; ALB, albumin; AM, aseptic meningitis; NA, not available; OCB, oligoclonal bands; pSS, primary Sjögren’s syndrome; SS, Sjögren’s syndrome

Reference	Age (years)	Sex	Pre-diagnosis SS	Neurological symptoms	Neurological diagnosis	Serum anti-SS-A/SS-B antibodies	CSF anti-SS-A/SS-B antibodies	CSF cells (n/μL)	CSF protein (mg/dL)	CSF glucose (mg/dL)	CSF ALB (mg/L)	ALB index	CSF IgG (mg/dL)	Serum IgG (mg/dL)	IgG index	AI	CSF OCB	CSF IL-6 (pg/mL)
Mégevand et al. (2007) [3]	45	F	SS diagnosed 14 years earlier	Paresthesia; progressive cognitive impairment	pSS-related CNS involvement	+ / +	+ / +	6 (93% lymphocytes)	NA	NA	207	6.2	25.3	3920	1.04	1.67	+	NA
	60	M	SS diagnosed several years earlier	Progressive chorea; cognitive dysfunction	pSS-related CNS involvement	+ / -	+ / -	1 (83% lymphocytes)	NA	NA	200	6.25	4.5	852	0.85	0.92	-	NA
	59	F	None	Paraparesis; hypoesthesia of both lower limbs; urinary incontinence	Myelitis	+ / -	+/-	73 (92% lymphocytes)	NA	NA	184	7.67	9	1400	0.84	4.29	+	NA
Kurotaki et al. (2022) [4]	18	F	Isolated SS-A/SS-B antibody positivity for seven years	Fever; headache; vomiting	AM	+ / +	+ / +	50 (93% polymorphonuclear cells)	79.3	64	NA	NA	14.9	2583	NA	NA	NA	2623
Butryn et al. (2020) [8]	39	F	None	Severe sensory ataxia; mild paraparesis	Myelitis	+ / NA	+ / NA	45	35.5	NA	NA	10.9	NA	NA	NA	2.2	+	NA
	50	F	SS diagnosed two years earlier	Slight sensory ataxia; dysesthesia	Myelitis	+ / NA	+ / NA	0	38	NA	NA	5	NA	NA	NA	1	-	NA
	52	M	None	Sensorimotor tetraparesis	Myelitis	+ / NA	+ / NA	39	46.2	NA	NA	6.9	NA	NA	NA	5.1	+	NA
Present case	59	F	None	Fever; headache; arthralgia	AM	+ / -	+ / -	179 (93.9% mononuclear cells)	96	39	595	12.7	13.1	1685.1	0.61	6.4	-	26.5

Including the patient in the present case, the eight patients were 18-60 years old, and 75% were female. SS diagnosis preceded the onset of neurological symptoms in 50% of cases. Myelitis, SS-associated CNS involvement, and AM were observed in 50%, 25%, and 25% of cases, respectively. Although our patient exhibited sicca symptoms before AM onset, pSS-associated CNS involvement can occur as the initial neurological presentation [3,8,9].

All patients were positive for both serum and CSF anti-SS-A antibodies, with CSF cell counts ranging from 0 to 179 cells/μL and protein levels from 35.5 to 96 mg/dL (Table 2). CSF oligoclonal bands were detected in four of the seven tested patients, and an elevated IgG index was observed in three of the four tested patients. The anti-SS-A antibody index was elevated in five of the seven tested patients (Table 2). In agreement with previous reports, our patient was positive for anti-SS-A antibodies in both serum and CSF and had compatible CSF findings.

Compared with previously reported cases of pSS-associated CNS involvement with CSF anti-SS-A antibody positivity, the present case is distinctive in several respects. First, marked hypoglycorrhachia (30 mg/dL) was observed, whereas CSF glucose levels were normal or not reported in earlier cases [3,4,8]. Second, the combination of an elevated CSF anti-SS-A antibody index, increased albumin quotient, and hypoglycorrhachia suggests a greater degree of blood-brain barrier disruption and immune-mediated inflammation than typically described. Third, steroid-responsive improvement with subsequent CSF glucose normalization provides therapeutic insight not emphasized in prior reports. Collectively, these findings expand the clinical spectrum of pSS-associated AM and support consideration of immune-mediated etiologies even when hypoglycorrhachia suggests infection.

The elevated CSF anti-SS-A antibody index suggests intrathecal immune activation rather than passive diffusion from serum, while the increased albumin quotient indicates blood-brain barrier disruption. Although hypoglycorrhachia is most commonly associated with infectious meningitis, we hypothesize that intense immune-mediated meningeal inflammation and blood-brain barrier dysfunction may reduce CSF glucose levels through altered glucose transport and increased metabolic demand. This mechanistic interpretation remains hypothetical and is supported by clinical correlation rather than direct experimental evidence. Whether anti-SS-A antibodies are directly pathogenic or instead serve as surrogate markers of broader immune activation in the CNS remains unclear and warrants further investigation.

From a diagnostic perspective, hypoglycorrhachia typically raises concern for infectious, neoplastic, or neurosarcoid meningitis [6,10]. CSF cytology was not suggestive of malignant meningitis, and no radiological findings supported neoplastic involvement. Although neurosarcoidosis is the most frequent noninfectious immune-mediated cause of low CSF glucose, our patient exhibited no systemic sarcoid features, and negative microbiological studies excluded infection. Notably, the steroid-responsive improvement in both symptoms and CSF findings further supports an immune-mediated mechanism. Accordingly, pSS-associated AM should be considered in patients with unexplained hypoglycorrhachia when autoimmune features and CSF anti-SS-A antibodies are present.

This report has some limitations. As a single case report, the findings have limited generalizability, and causal relationships cannot be established. Therefore, the observed association between decreased CSF glucose levels and pSS-associated AM should be interpreted as hypothesis-generating.

## Conclusions

In patients with AM of unknown etiology, evaluation of autoimmune features and measurement of serum and CSF anti-SS-A antibodies may facilitate diagnosis. Reduced CSF glucose levels may serve as a supportive finding in SS-associated AM.
